# Glances at the Hospitals

**Published:** 1903-05-16

**Authors:** 


### GLANCES AT THE HOSPITALS.

### STAMFORD AND RUTLAND INFIRMARY.

The income amounted to £2,948 and the expenditure to £2,711, and there was a balance due to treasurer of £694 on January 1st, 1902. In the general wards the number of in-patients was 271, and in the isolation wards 71. The daily average number in residence was 28, and the average stay in the hospital general wards was 20 days, and in the isolation wards 43 days. Of the total number in the general wards 132 were discharged cured, 65 relieved, 7 incurable, 9 for other reasons, 30 were made out-patients, and 11 died. Of the 71 cases admitted to the isolation wards, 65 were discharged cured, 2 died, and 4 remained under treatment. The medical and surgical tables are fairly well compiled. Of 49 cases of scarlet fever 48 recovered and 1 was under treatment, of 10 cases of enteric fever 9 recovered and 1 was under treatment, of 7 cases of diphtheria all recovered. Among the surgical cases we note that of 9 cases of hernia 5 were cured and 4 relieved. Of 9 cases of appendicitis 3 were cured, 2 relieved, and 4 died. We regret that the operation table does not show the results. An anæsthetic table tells us that ether was given 30 times, chloroform 40 times, A.C.E. mixture 4 times, ether and A.C.E. mixture twice, and gas and ether 14 times. On page 13 a useful table is given. It shows the mortality among the infectious diseases during the last 23 years, and we find that the mortality from enteric fever has been 5 per cent., that from scarlet fever 1·8 per cent., and from diphtheria 10 per cent.

### VICTORIA HOSPITAL, BLACKPOOL.

The income for the year was £1,674, and there was an adverse balance of £194 on December 31st, part of this having been carried over from the previous year. The first complete year of the existence of this hospital was 1895, and during that year it had 69 in-patients and 34 out-patients, whereas last year the numbers were respectively 394 and 1,362. It is now proposed to enlarge the buildings. On January 1st there were 32 patients in residence; the average daily number of beds occupied was 33, and the average stay in the hospital was 31 days. In the previous year it was 33 days, and we think that a still further decrease is possible. The medical and surgical tables are carefully drawn out, and give all necessary information concerning the cases; 116 operations were performed. We did not notice any table showing the nature of anæsthetics used.

### SWANSEA GENERAL HOSPITAL.

The total ordinary income was £6,150, and the extraordinary amounted to £232. The ordinary expenditure was £5,235, so that there was a substantial balance on the right side. It is most satisfactory to be able to state that the workpeople's subscriptions have increased from £1,617 to £2,193. The hospital ball brought in £252. Several large donations have been received including two of £5,000 each. The Board have decided to build a convalescent home at an estimated expense of £4,047; and the private nursing arrangements are to be developed. The total number of in-patients treated during the year was 1,176. The daily average of beds occupied was 94, and the average stay in the hospital was 29 days. There were 77 deaths. The total number of operations performed was 1,127. Of these 667 were in the general in-patients' department, 183 in the eye department, and 277 in the out-patients' and casualty department. These bald figures are practically all that can be extracted from the medical report, and they tell us nothing about the diseases from which the 1,176 in-patients suffered, and nothing about the results of treatment.

### BRISTOL ROYAL HOSPITAL FOR WOMEN AND CHILDREN.

The ordinary income amounted to £3,972, and the expenditure to £4,825, showing an adverse balance of £853. The governors state that the expenses at their convalescent home have increased, and the commissariat and domestic departments have cost more than in previous years. These facts show that another £800 a year must be raised to prevent the institution drifting into debt. The committee very properly expected that more help might have been looked for from the workpeople themselves, and on referring to the balance-sheet we find that only £192 have been received from this source, a sum which ought certainly to be largely increased in the future. During the year 685 children and 64 women have been admitted as in-patients, and 3,213 children and 1,899 women have been treated as out-patients, involving 24,681 attendances. These figures prove that much useful work is being done by the hospital, and that it deserves the support of all classes. We extract the above particulars from an advanced proof sheet of the report of the committee, consequently we cannot at present refer to any medical or surgical details.

### STRATFORD-UPON-AVON HOSPITAL.

The ordinary receipts were £1,365, being a slight increase on those of last year. The expenditure amounted to £1,396, showing a deficiency of £31. This deficit added to that of the previous year amounted to a total of £161. The operating theatre has been enlarged and refurnished at an expense of £500, and an ophthalmic surgeon has been appointed. At the beginning of the year there were 17 patients in residence, and 175 were admitted during the year, making a total of 192 under treatment. Of these 119 were cured, 33 relieved, 6 incurable, 11 were discharged for various reasons, 8 died, and 15 remained in the hospital. The out-patients numbered 342.

